# Supplementation of Miso to a Western-Type Diet Stimulates ILC3s and Decreases Inflammation in the Small Intestine

**DOI:** 10.3390/nu16213743

**Published:** 2024-10-31

**Authors:** River Budau, Takuro Okamura, Yuka Hasegawa, Naoko Nakanishi, Masahide Hamaguchi, Michiaki Fukui

**Affiliations:** Department of Endocrinology and Metabolism, Graduate School of Medical Science, Kyoto Prefectural University of Medicine, Kyoto 602-8566, Japan; riverbudau@gmail.com (R.B.);

**Keywords:** inflammation, innate lymphoid cells, miso, short-chain fatty acids, small intestine, Western-type diet

## Abstract

Background/Objectives: Western-type diets (WDs) damage the intestinal barrier by disrupting the gut microbiota composition and causing inflammation, leading to the development of obesity, type 2 diabetes, and non-alcoholic fatty liver disease. Short-chain fatty acids (SCFAs) are produced by the gut microbiota and found in fermented foods and can stimulate the anti-inflammatory action of type 3 innate lymphoid cells (ILCS3s) in the intestine. This study hypothesised that supplementing miso, a Japanese fermented food, to a WD could increase the levels of SCFAs and thus stimulate ILC3s, decreasing inflammation in the intestine and protecting intestinal barrier integrity. Methods: Mice with RORγt total (KI/KI) or partial (KI/w) knockout were fed a high-fat high-sugar diet (HFHSD) for eight weeks as a model of WD. Half of the mice received miso supplementation in addition to the HFHSD. Weight gain, glucose tolerance and insulin resistance, intestinal barrier integrity, intestinal immunity, and liver condition were assessed. Results: Miso supplementation increased SCFA levels in the small intestine, which stimulated ILC3 function in KI/w mice. Glucose tolerance was improved, intestinal barrier integrity was ameliorated, and mucus production was increased. The level of IL-22 was increased, while pro-inflammatory ILC1s, M1 macrophages, TNF-α, and IL-1β were decreased. Liver condition was not affected. Conclusions: This study demonstrated that miso supplementation influenced several factors involved in inflammation and intestinal barrier integrity by stimulating ILC3s in RORγt heterozygous mice. Moreover, it showed that the number of ILC3s is not the key factor in immune regulation, but rather the ability of ILC3 to produce IL-22 and employ it to control the immune response in the small intestine.

## 1. Introduction

Western-type diets (WDs) are composed of high amounts of fats and sugars, mainly derived from processed foods, while they lack necessary nutrients such as fibres, vitamins, and minerals [1,2]. The consumption of WDs has been increasing in the last decades and has been associated with a rise in incidence of disorders such as obesity, type 2 diabetes, and non-alcoholic fatty liver disease [3,4]. On the other hand, traditional dietary patterns such as the Mediterranean diet and the Japanese diet have a varied composition and are predominantly composed of unprocessed foods [5,6]. Moreover, traditional diets are correlated with good metabolic health and low inflammation [5].

The intestinal barrier is composed of epithelial cells, a mucus layer, the gut microbiota, and immune cells [7,8]. The gut microbiota plays an important role in food processing and produces short-chain fatty acids (SCFAs) in the presence of dietary fibre. SCFAs are crucial for the maintenance of the intestinal barrier integrity, but their levels are decreased when the microbiota composition is disrupted, a state known as dysbiosis [9,10]. Damage to the intestinal barrier can result in chronic inflammation, which subsequently contributes to the onset of metabolic disorders [11,12].

Unbalanced diets such as WDs have been correlated with dysbiosis and dysbiosis-related disorders, while balanced diets contribute to the maintenance of a rich and varied gut microbiota composition [2,11]. Notably, fermented foods are known to positively impact the gut microbiota by supplementing commensal microbes and providing nutrients for the existing microbes. Additionally, certain fermented foods directly provide SCFAs [10,13]. The Japanese traditional diet is especially rich in fermented foods, mainly soybean products such as miso, soy sauce, and natto [14,15]. Consumption of these fermented foods has been reported to affect gut microbiota composition and to increase the levels of SCFAs in addition to preventing metabolic and inflammatory disorders [16,17].

Innate lymphoid cells (ILCs) are tissue-resident cells involved in mucosal immunity, and they are divided into three categories: ILC1s, ILC2s, and ILC3s [18]. ILC3s are abundant in the intestine, where their main roles are maintaining tissue homeostasis and regulating immune response. ILC3 activity is mediated by the production of interleukin (IL-22), which maintains homeostasis and promotes the proliferation of intestinal stem cells, and IL-17, which stimulates the secretion of chemokines and chemoattractants and recruits pro-inflammatory neutrophils [19]. ILC3 function is enhanced by the presence of SCFAs, which induce the production of IL-22 and its anti-inflammatory action [20].

In the present study, we hypothesised that supplementation of fermented foods causes an increase in SCFAs in the intestine, which stimulates ILC3-mediated IL-22 production and improves inflammation and maintains barrier integrity. This study aimed to investigate the effects of miso, a fermented food, on ILC3s by using RORγt^gfp/gfp^ and RORγt^gfp/wt^ mice fed a WD.

## 2. Materials and Methods

### 2.1. Animals

The experimental procedures in this study were approved by the Committee for Animal Research at the Kyoto Prefectural University of Medicine. ROR(γt)-EGFP male mice were acquired from the Jackson Laboratory (007572; Bar Harbor, ME, USA) and employed for this study [21,22]. RORγt^gfp/gfp^ mice (homozygous) are referred to as RORγt KI/KI mice, while RORγt^gfp/wt^ mice (heterozygous) are referred to as RORγt KI/w mice. The functions of ILC3s are impaired in RORγt KI/KI mice, while they are comparable to wild-type mice in RORγt KI/w mice [23].

All mice were fed a high-fat, high-sucrose diet (HFHSD; 459 kcal/100 g, 20% protein, 40% carbohydrate, and 40% fat; D12327, Research Diets, Inc., New Brunswick, NJ, USA) from 8 weeks of age until 16 weeks. Feed and water were supplied in equal amounts for pair feeding. Additionally, miso (FUNDOKIN Co., Ltd., Oita, Japan) was administered ad libitum by mixing miso paste with water (48 g miso, 200 mL water). The miso paste used in this study contained 191 kcal/850 g and was determined not to impact the energy intake of the mice [24].

**Figure 1:**
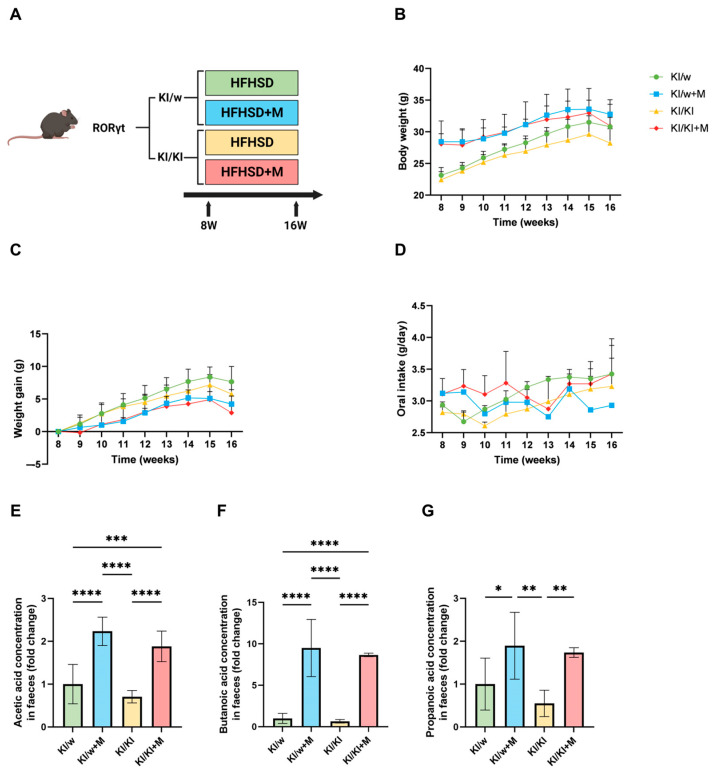
Study design, changes in body weight, weight gain, and food intake, and concentration of SCFAs. (**A**) Administration of HFHSD or HFHSD+M started at 8 weeks of age; mice were sacrificed at 16 weeks of age. (**B**) Body weight changes from week 8 to 16 (*n* = 6). (**C**) Weight gain (*n* = 6). (**D**) Food intake changes (*n* = 6). Concentration of (**E**) acetic acid, (**F**) butanoic acid, and (**G**) propanoic acid in faeces of 16-week-old mice (*n* = 6). Data are represented as the mean ± SD values; data were analysed using a one-way ANOVA with a Holm–Šídák multiple-comparison test; * *p* < 0.05, ** *p* < 0.01, *** *p* < 0.001, and **** *p* < 0.0001.

Twenty-four mice were divided into the following groups: (I) RORγt KI/w fed HFHSD, (II) RORγt KI/w fed HFHSD and miso (HFHSD+M), (III) RORγt KI/KI fed HFHSD, and (IV) RORγt KI/KI fed HFHSD+M. As the KI/w phenotype possesses functioning ILC3s and is comparable to wild-type mice, the first group (KI/w fed HFHSD) was used as control. Body weight of the mice was measured twice a week and oral intake was monitored. At 16 weeks of age, all mice were euthanised by exposure to lethal anaesthesia (4.0 mg/kg midazolam, 0.3 mg/kg medetomidine, and 5.0 mg/kg butorphanol) after an overnight fast (Figure 1A) [25].

### 2.2. Measurement of Fatty Acid Levels

Fatty acids were extracted from faecal samples taken from the large intestine. Rectal faeces (20 mg) were mixed with 500 µL of distilled water and 500 µL of acetonitrile, then ground in a ball mill at 4000 rpm for 2 min. The resulting mixtures were placed in a shaker at 1000 rpm for 3 min at 37 °C, followed by centrifugation for 3 min at 14,000 rpm at room temperature. The supernatant (500 µL) was mixed with 500 µL of acetonitrile, and then shaken and centrifuged again. The final samples (500 µL) were mixed with 500 µL of ACFS water. The pH level of all samples was adjusted to 8.0 using NaOH. Blank solution and standard solution were prepared according to the manufacturer’s directions.

The concentration of SCFAs was determined by gas chromatography/mass spectrometry (GC/MS) using Agilent 7890B/7000D system (Agilent Technologies; Santa Clara, CA, USA). Samples were loaded into a Varian capillary column (DB-FATWAX UI; Agilent Technologies), which was maintained at 100 °C for 4 min, after which the temperature was then gradually increased at a rate of 3 °C per minute until reaching 240 °C, and held stable for 7 min. The samples were injected in split mode with a split ratio of 5:1, and each fatty acid methyl ester was detected using the selected ion-monitoring mode. Results were normalised to the peak height for the C17:0 internal standard [26].

### 2.3. Analytical Procedures for Glucose and Insulin Tolerance Tests

Intraperitoneal glucose tolerance test (iPGTT) and insulin tolerance test (ITT) were performed after fasting the mice for 16 h and 5 h, respectively. The tests were performed on all mice on the week before sacrifice, with a one-day interval between iPGTT and ITT. Blood samples were collected via the tail vein and glucose levels were measured using a glucometer (FS Precision Blood Glucose Measurement Electrode; Abbot Park, IL, USA) at specific time points (0, 30, 60, and 120 min after injection for iPGTT; 0, 15, 30, 60, and 120 min for ITT). The area under the curve (AUC) of the iPGTT and ITT results was analysed.

### 2.4. Blood Biochemistry

Blood samples were collected from fasted mice by cardiac puncture during euthanasia, and serum was attained by centrifugating at 14,000 rpm for 10 min at 4 °C. Alanine transaminase (ALT) levels were measured following the standardization method outlined by the Japanese Society for Clinical Chemistry, as carried out in previous studies. Triglycerides (TGs) and non-esterified fatty acids (NEFAs) were measured via enzymatic methods as outlined in previous studies [26]. All biochemical examinations were performed by FUJIFILM Wako Pure 18 Chemical Corporation (Osaka, Japan).

### 2.5. Histology of Small Intestine and Large Intestine

Tissue from the small intestine (jejunum) was obtained and fixed with 10% buffered formaldehyde, embedded in paraffin, cut into 4 µm thick sections, and stained with haematoxylin and eosin (H&E). Large intestine (colon) tissue was fixed in Carnoy’s solution, processed, and stained with periodic acid–Schiff (PAS). Images were captured with a fluorescence microscope (BZ-X719, Keyence, Itasca, IL, USA) and analysed using ImageJ software (version 1.53k; NIH; Bethesda, MD, USA). The height and width of the villi and the crypt depth in the jejunum were analysed and measured at five locations per slide for each group using ImageJ software. Goblet cells were counted and reported as the average number of cells per ten crypts using ImageJ software.

### 2.6. Isolation of Mononuclear Cells from Small Intestine and Liver

Systemic perfusion with heparinised saline was performed before harvesting small intestine and liver tissue in order to prevent blood contamination of the samples. The samples were stored in 2% Foetal Bovine Serum (FBS) and isolation of mononuclear cells was performed. The Lamina Propria Dissociation Kit (10-097-410; Miltenyi Biotec; San Diego, CA, USA) was used to isolate intestinal lamina propria leukocytes (LPL) mononuclear cells from small intestine. LPL mononuclear cells were obtained by washing twice with Roswell Park Memorial Institute (RPMI) 1640 medium containing 2% FBS.

The liver was harvested and hepatic lymphocytes were isolated through mechanical dissection. Liver tissue was filtered and then suspended in RPMI + 2% FBS. After centrifugation at 1600 rpm, cell pellets were resuspended in 40% Percoll^®^ (Sigma-Aldrich; St. Louis, MO, USA) and slowly added to a tube containing an equal volume of 60% Percoll^®^. Cells were aspirated from the Percoll interface and centrifuged, and the pellet was washed twice with RPMI + 2% FBS and centrifuged before use.

### 2.7. Flow Cytometry

The cell suspensions were stained as in previous studies [26]. Staining of innate lymphoid cells was performed using the following antibodies: Biotin-CD3e (Biolegend 100304; clone: 145-2C11; 1:200 dilution), Biotin-CD45R/B220 (Biolegend 103204; RA3-6B2; 1:200), Biotin-Gr-1 (Biolegend 108404; RB6-8C5; 1:200), Biotin-CD11c (Biolegend 117304; N418; 1:200), Biotin-CD11b (Biolegend 101204; M1/70; 1:200), Biotin-Ter119 (Biolegend 116204; clone: TER-119; 1:200), Biotin-FceRIa (Biolegend 134304; MAR-1; 1:200), FITC-Streptavidin (Biolegend 405202; 1:500), PE-Cy7-CD127 (Biolegend 135014; A7R34; 1:100), Pacific Blue-CD45 (Biolegend 103116; 30-F11; 1:100), PE-GATA-3 (Invitrogen; TWAJ; 1:50), APC-RORγ (Invitrogen; AFKJS-9; 1:50), and Fixable Viability Dye eFluor 780 (Invitrogen; 1:400) (Figure S1). Staining of M1 and M2 macrophages was performed using the following antibodies: APC-CD45.2 (Invitrogen 12480182; 104; 1:50), APC-Cy7-CD11b (Biolegend 47011282; M1/70; 1:50), FITC-CD206 (Invitrogen MA516870; MR5D3; 1:50), and PE-Cy7-CD11c (Invitrogen 25011482; N418; 1:50) (Figure S2). The stained cells were analysed with FACS Canto II, and the results were analysed using FlowJo version 10 (TreeStar; Ashland, OR, USA).

### 2.8. Gene Expression Analysis in Small Intestine

Small intestines of mice were excised and frozen in liquid nitrogen. The samples were then homogenised in QIAzol Lysis reagent (Qiagen; Venlo, The Netherlands), and total RNA was isolated according to the manufacturer’s directions. Total RNA (0.5 µg) was reverse-transcribed by employing a High-Capacity cDNA Reverse Transcription Kit (Applied Biosystems; Foster City, CA, USA) for first-strand cDNA synthesis, using an oligonucleotide dT primer and random hexamer priming in accordance with the manufacturer’s instructions. The reverse transcription reaction was carried out for 120 min at 37 °C, followed by inactivation of reverse transcription for 5 min at 85 °C.

The mRNA expression levels of *Il22* (Mm01226722_g1, ThermoFisher Scientific, MA, USA), *Tnfa* (Mm00443258_m1), *Il1b* (Mm00434228_m1), *Ifng* (Mm01168134_m1), *Muc2* (Mm01276676_m1), *Pept1* (Mm04209483_m1), and *Sglt1* (Mm00451203_m1) were quantified in the small intestine via real-time reverse transcription polymerase chain reaction (RT-PCR). The PCR conditions were as follows: incubation at 50 °C for 2 min and at 95 °C for 20 s, and then 40 cycles at 95 °C for 1 s and at 60 °C for 20 s. The relative expression levels of target genes were normalised to threshold cycle (CT) values for *Gapdh* and quantified using the comparative threshold cycle 2^−∆∆CT^ method. The signals obtained for the KI/w group (control) were assigned to the relative value of 1.0.

### 2.9. Histology of Liver

Liver tissue was fixed in either 10% buffered formaldehyde or 4% paraformaldehyde buffered solution and stained with H&E, Masson’s Trichrome (MT), and Oil Red, as detailed in previous studies [26]. Images were captured using a fluorescence microscope (BZ-X719, Keyence). H&E- and MT-stained liver sections were used to assess the extent of the liver damage. The NAFLD activity score was employed in order to assess the severity of NAFLD [27]. The scoring system is comprised of 14 features, with 4 evaluated semi-quantitatively: steatosis, lobular inflammation, hepatocellular ballooning, and fibrosis. Additionally, fibrosis was assessed as follows: none (0); mild, zone 3, perisinusoidal (1A); moderate, zone 3, perisinusoidal (1B); portal/periportal (1C); perisinusoidal and portal/periportal (2); bridging fibrosis (3); and cirrhosis (4). Furthermore, the Oil-Red-stained area of the liver sections was calculated using ImageJ software.

### 2.10. Statistical Analysis

The data were analysed using GraphPad Prism software (Version 9.5.0; San Diego, CA, USA). One-way analysis of variance (ANOVA) with Holm–Šídák multiple-comparison test was applied to compare the four groups. Statistical significance was set at *p* < 0.05. Figures were generated using GraphPad Prism software.

## 3. Results

### 3.1. Body Weight and Oral Intake Were Monitored During the Treatment with HFHSD or HFHSD+M

The body weights of the mice and oral intake of the HFHSD were measured biweekly, while miso supplementation was administered ad libitum to the KI/w+M and KI/KI+M groups. No significant difference was observed in the body weights at the end of the study between the four groups (Figure 1B). However, as the initial body weight was not the same between groups, the weight gain throughout the treatment was calculated. In this case, the two groups that did not receive miso supplementation had a higher weight gain, although the difference was not significant (Figure 1C). Additionally, the oral intake was calculated and showed no significant difference (Figure 1D).

### 3.2. SCFA Concentration in the Intestine Was Increased by Miso Supplementation

In order to determine if the miso supplementation was successful in increasing the levels of SCFAs in the small intestine, the concentration of acetic acid, butanoic acid, and propanoic acid was quantified in faecal samples obtained from the large intestine. In all three cases, the concentration was significantly increased in KI/w+M mice compared to KI/w, with no significant difference between the KI/w and KI/KI groups, nor between the KI/w+M and KI/KI+M groups (Figure 1E–G). The increase of SCFAs in the intestine can be attributed to the high amount of SCFAs contained in the miso compared to the HFHSD, which lacks the fibres and microbes necessary for the fermentation process. Additionally, miso consumption can increase the production of SCFAs in the intestine by providing dietary fibre and by supplementing commensal microbes to the pre-existing gut microbiota. In order to understand at which stage the SCFA increase occurred (in the food or in the intestine), SCFA concentration in the miso and sequencing of the gut microbiota are required. However, due to time constraints and technical limitations, these analyses were not performed as part of the study.

### 3.3. Glucose Tolerance Was Increased but Insulin Resistance Was Unaffected by Miso Supplementation in KI/w Mice

**Figure 2:**
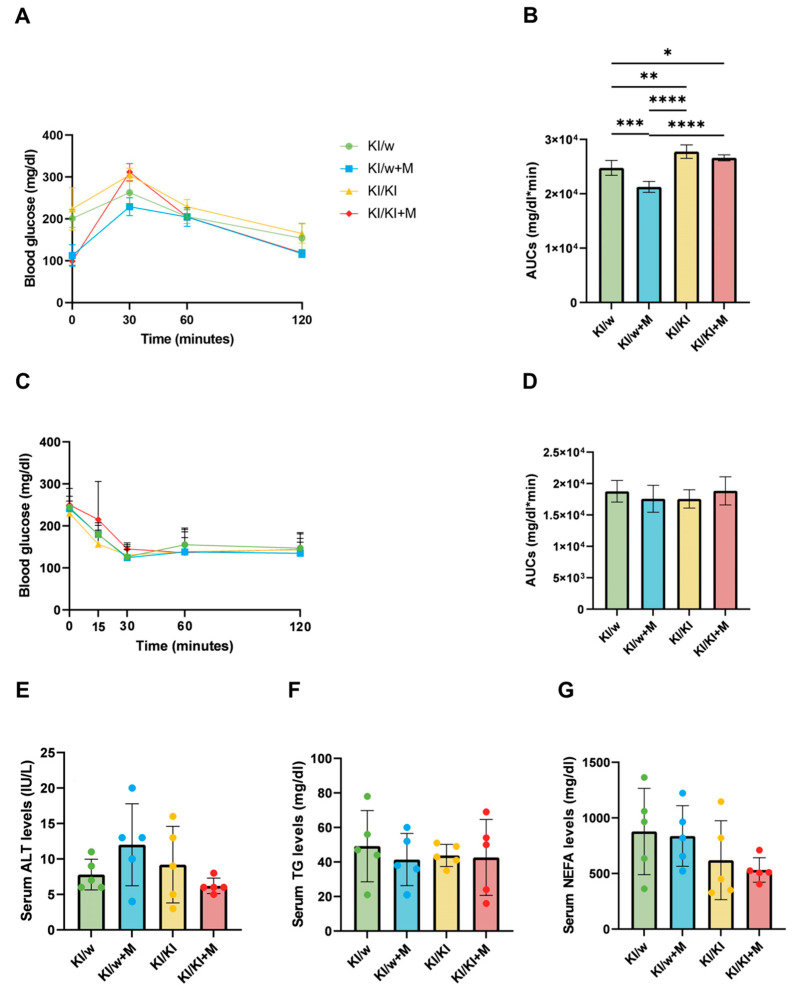
Results of iPGTT and ITT and blood biochemistry. (**A**,**B**) Results of intraperitoneal glucose tolerance testing (iPGTT; 1g/kg of body weight) and area under the curve (AUC) analysis in 15-week-old mice (*n* = 6). (**C**,**D**) Results of insulin tolerance testing (ITT; 0.5 U/kg) and AUC analysis in 15-week-old mice (*n* = 6). Serum levels of (**E**) ALT, (**F**) TG, and (**G**) NEFAs in 16-week-old mice (*n* = 6). Data are represented as the mean ± SD values; data were analysed using a one-way ANOVA with a Holm–Šídák multiple-comparison test; * *p* < 0.05, ** *p* < 0.01, *** *p* < 0.001, and **** *p* < 0.0001.

Glucose tolerance and insulin tolerance were assessed by performing iPGTT and ITT on the week before sacrifice, and the AUC of each group was calculated and plotted (Figure 2A,C). These factors are not directly regulated by ILC3s, but they can be a consequence of chronic inflammation and thus they can be affected by ILC3 function in the small intestine. The iPGTT results showed that blood glucose was significantly decreased in KI/w mice treated with miso as opposed to those without miso, and that it was significantly increased in the KI/KI+M group compared to the KI/w+M group with no difference between KI/KI and KI/KI+M (Figure 2B). This indicates that the glucose tolerance of the KI/w+M group was improved by the miso supplementation in the presence of functioning ILC3s, despite the presence of the HFHSD. However, the ITT results showed no significant difference between the four groups and thus the insulin resistance was unaffected (Figure 2D).

### 3.4. Serum Metabolic Markers Were Not Affected by Miso Supplementation

In order to observe the overall state of metabolic dysregulation caused by the HFHSD, the levels of alanine transaminase (ALT), triglycerides (TGs), and non-esterified fatty acids (NEFAs) in the serum were quantified as markers of metabolic disorder. In particular, ALT is employed as a marker of liver damage, TGs as a marker of cardiovascular risk, and NEFAs as a marker of insulin resistance, which are all associated with the fat accumulation and increased inflammation caused by the HFHSD [28,29,30]. However, no difference was found between the four groups, although results were varied between mice of the same group (Figure 2E–G).

### 3.5. Intestinal Barrier Integrity Was Protected by Miso Supplementation in KI/w Mice

**Figure 3:**
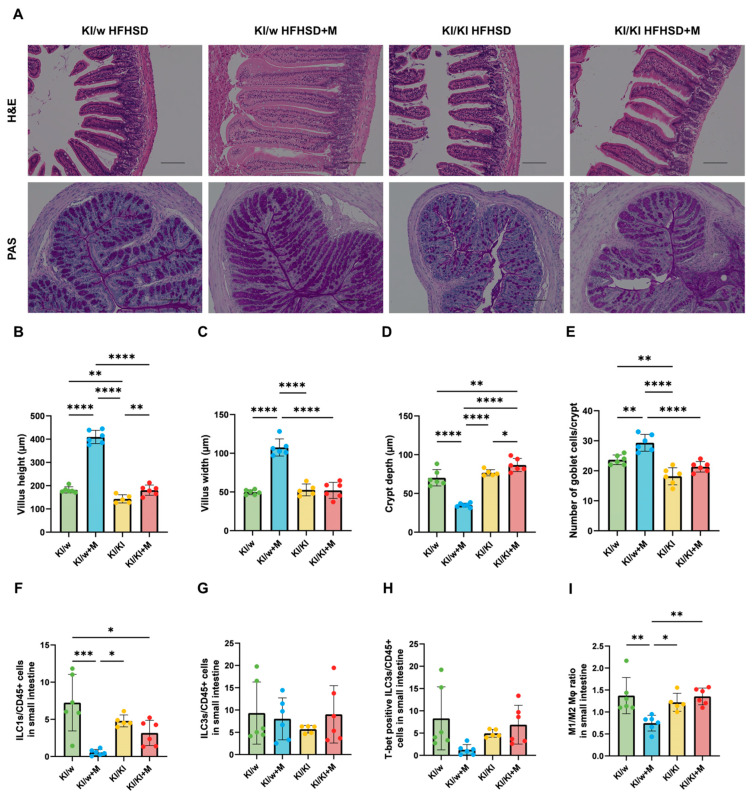
Histological evaluation of the small and large intestines and immune cells in the small intestine. (**A**) Representative images of haematoxylin and eosin (H&E)-stained jejunum and periodic acid–Schiff (PAS)-stained colon sections, collected at 16 weeks of age. The scale bar represents 100 μm. (**B**) Villus height, (**C**) villus depth, and (**D**) crypt depth in the jejunum (*n* = 6). (**E**) Total goblet cells per crypt in the colon (*n* = 6). (**F**) Ratio of ILC1s to CD45-positive cells (*n* = 6). (**G**,**H**) Ratios of ILC3s to CD45-positive cells and of T-bet positive ILC3s to CD45-positive cells (*n* = 6). (**I**) Ratio of M1 to M2 macrophages (*n* = 6). Data are represented as the mean ± SD values; data were analysed using a one-way ANOVA with a Holm–Šídák multiple-comparison test; * *p* < 0.05, ** *p* < 0.01, *** *p* < 0.001, and **** *p* < 0.0001.

The small intestines of all of the mice were sampled and analysed to assess the effects of the two diets on the KI/w and KI/KI mice. Representative images of small intestine (jejunum) sections stained with H&E and of large intestine (colon) sections stained with PAS are shown in Figure 3A. In order to determine if intestinal barrier integrity was affected by the diet and mouse genotype, villus and crypt size were measured in the small intestine, and goblet cells were enumerated in the large intestine.

In the small intestine, a very significant increase was detected in the villus height and width of the KI/w+M mice compared to both KI/w and KI/KI+M groups (Figure 3B,C). Additionally, a very significant decrease was observed in the crypt depth of KI/w+M mice compared to the KI/w and KI/KI+M groups (Figure 3D). Since a big villus height and width are associated with better digestive and absorption capability, and a small crypt depth is associated with efficient tissue turnover [31,32], this shows that miso supplementation was able to preserve the intestinal barrier integrity in the presence of functioning ILC3s. In the large intestine, the ratio of goblet cells per crypt showed a significant increase in KI/w+M mice compared to both the KI/w and KI/KI+M groups (Figure 3E). This can be associated both with the positive role of ILC3s in small intestine homeostasis, and with the increase in mucin production induced by butanoic acid.

### 3.6. ILC Differentiation and Macrophage Polarisation in the Small Intestine Were Affected by Miso Supplementation in KI/w Mice

The ratios of ILC1s and ILC3s to CD45-positive cells, as well as the ratio of M1 to M2 macrophages, were assessed in the small intestine in order to evaluate the differentiation of immune cells and thus the inflammatory state within this region. The number of ILC1s was significantly decreased in KI/w mice treated with miso compared to KI/w without miso, while no significant difference was observed between KI/KI mice treated with or without miso. This indicates that the pro-inflammatory function of ILC1s was countered by the miso supplementation in the presence of functioning ILC3s, as no significant difference was shown between the KI/w+M and KI/KI+M groups (Figure 3F). No significant difference was observed in the levels of ILC3s and T-bet positive ILC3s in the four groups (Figure 3G,H). However, this does not indicate whether there is a difference in the function of ILC3s. Additionally, macrophage polarisation presented an anti-inflammatory phenotype in the KI/w+M group compared to the other three groups, as the ratio of M1 to M2 macrophages was significantly decreased, while no difference was observed between the KI/KI and KI/KI+M mice (Figure 3I).

### 3.7. Pro- and Anti-Inflammatory Cytokine Production in the Small Intestine Was Affected by Miso Supplementation

The expression of genes encoding for pro- and anti-inflammatory cytokines was quantified and the fold change was calculated in order to observe the difference in immune regulation in the small intestine. In particular, ILC quantification with flow cytometry was not sufficient to determine the difference in the function of ILC3 caused by RORγt knockout and SCFA stimulation. For this reason, the effects of ILC3 on cytokine production were investigated.

**Figure 4:**
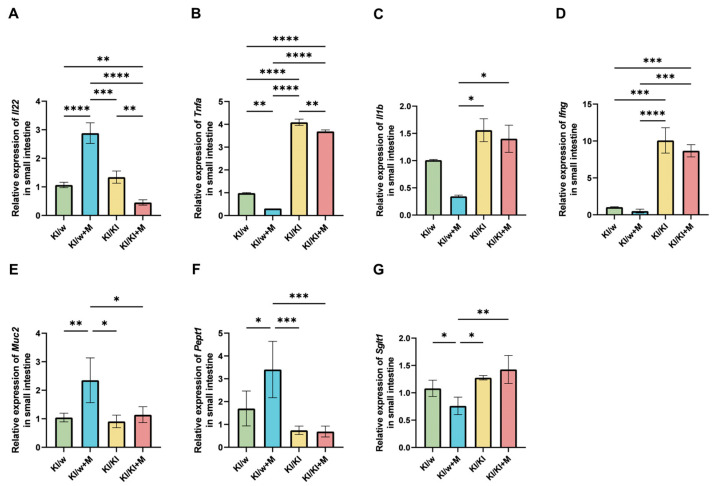
Gene expression of genes involved in inflammation, mucus production, and lipid metabolism in the small intestine. Relative mRNA expression of (**A**) IL-22, (**B**) Tnfa, (**C**) Il1b, and (**D**) Ifng normalised to the expression of Gapdh in 16-week-old mice (n = 2 or 3 each). Relative mRNA expression of (**E**) Muc2, (**F**) Pept1, and (**G**) Sglt1 normalised to the expression of Gapdh in 16-week-old mice (n = 2 or 3 each). Data are represented as the mean ± SD values; data were analysed using a one-way ANOVA with a Holm–Šídák multiple-comparison test; * *p* < 0.05, ** *p* < 0.01, *** *p* < 0.001, and **** *p* < 0.0001.

A significant increase in the expression of *IL-22* in KI/w mice treated with miso was observed compared to the KI/w and KI/KI+M groups (Figure 4A). Since IL-22 acts as the mediator of the anti-inflammatory phenotype of ILC3s, its increase indicates a heightened activity of ILC3s as a consequence of miso supplementation despite the number of ILC3s being unchanged between the four groups of mice. Additionally, a significant decrease in the expression of *Tnfa* was observed in KI/w+M mice compared to the other groups, indicating a decrease in inflammation in the small intestine (Figure 4B). However, the expression of *Tnfa* was significantly increased in the KI/KI and KI/KI+M groups compared to the KI/w and KI/w+M groups, indicating that the genotype had an impact in addition to the miso treatment. The expression of *Il1b* was decreased in KI/w+M mice compared to the double knockout groups, although no significant difference was found compared to the KI/w group. Nevertheless, there was no significant difference between KI/KI and KI/KI+M mice (Figure 4C). The expression of *Ifng* presented a much higher level in the KI/KI and KI/KI+M groups compared to KI/w and KI/w+M, similarly to *Tnfa*. However, in this case there was no difference between KI/w and KI/w+M mice (Figure 4D).

The expression of some additional genes was analysed. The expression of *Muc2* was significantly increased in the KI/w+M group compared to KI/w and KI/KI+M, with no difference between KI/KI and KI/KI+M (Figure 4E). As *Muc2* is responsible for the production of mucin, this result can be associated with the positive outcomes of the miso supplementation observed in the histological samples of the small and large intestines. The expression of *Pept1* was significantly increased in the KI/w+M mice compared to the KI/w and KI/KI+M groups, while no difference was present between the KI/KI and KI/KI+M groups (Figure 4F). This result indicates better absorption of peptides as a consequence of miso supplementation dependent on ILC3 function [33]. Finally, a significant decrease in *Sglt1* was found in the KI/w+M group as opposed to KI/w and KI/KI+M, with no significant difference between KI/K and KI/K+M (Figure 4G). *Sglt1* is responsible for glucose reabsorption and is known to be increased by T2D, which leads to hyperglycaemia [34]. Therefore, this result shows that miso supplementation is also beneficial against excessive glucose reabsorption in the small intestine.

### 3.8. Liver Damage and Weight Were Not Affected by Miso Supplementation

**Figure 5:**
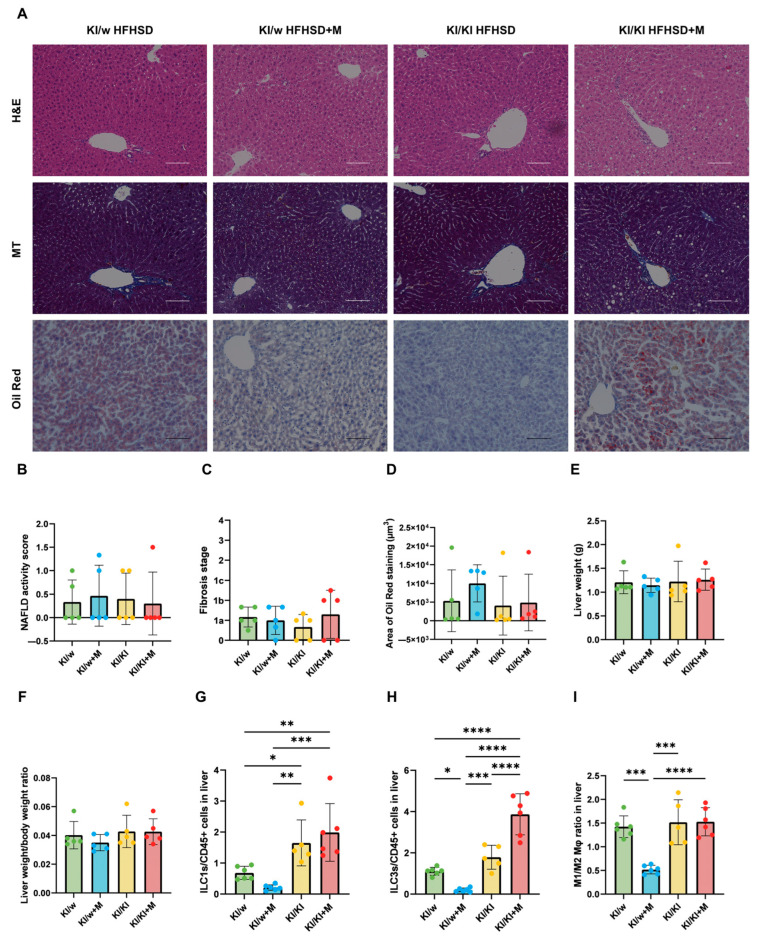
Histological evaluation of livers, liver weights, and immune cells in the liver. (**A**) Representative images of haematoxylin and eosin (H&E)-stained, Masson’s Trichome (MT)-stained, and Oil-Red-stained liver sections. Tissue was collected at 16 weeks of age. The scale bar represents 100 μm. (**B**,**C**) NAFLD activity score and fibrosis stage (*n* = 6). (**D**) Oil-Red-stained area (*n* = 6). (**E**) Liver weight at 16 weeks (*n* = 6). (**F**) Ratio of liver weight to body weight at 16 weeks (*n* = 6). (**G**) Ratio of ILC1s to CD45-positive cells (*n* = 6). (**H**) Ratio of ILC3s to CD45-positive cells (*n* = 6). (**I**) Ratio of M1 to M2 macrophages (*n* = 6). Data are represented as the mean ± SD values; data were analysed using one-way ANOVA with Holm–Šídák multiple-comparison test; * *p* < 0.05, ** *p* < 0.01, *** *p* < 0.001, and **** *p* < 0.0001.

In addition to the small intestine, the livers of all mice were sampled and analysed, as ILC3s are known to be abundant in this tissue. Representative images of liver sections stained with H&E, MT, and Oil Red are shown in Figure 5A.

In order to determine liver damage, the NAFLD score was calculated and the fibrosis stage was assessed. No significant difference was recorded in the liver damage between the groups, as the NAFLD and fibrosis scores were low in all of the mice (Figure 5B,C). Similarly, the Oil-Red-stained area showed no significant difference, indicating no difference in lipid accumulation in the liver (Figure 5D).

Additionally, the liver weights and the ratio of liver weight to body weight revealed no significant difference among the four groups (Figure 5E,F).

### 3.9. ILC Differentiation and Macrophage Polarisation in the Liver Were Affected by Miso Supplementation in KI/w Mice

The ratios of ILC1s and ILC3s to CD45-positive cells and that of M1 to M2 macrophages were assessed in the liver in order to determine if the miso supplementation had the same effect as observed in the small intestine. The number of ILC1s was increased in the KI/KI+M group compared to the KI/w+M group and in the KI/KI group compared to the KI/w group, but no difference was observed between the same genotype treated with or without miso (Figure 5G). In this case, the genotype of the mice affected the ILC1 distribution more than the diet. Additionally, the number of ILC3s showed a decrease in ILC3s in KI/w+M compared to the other groups (Figure 5H). This differed from the results in the small intestine, which had shown no change in the number of ILC3s. Finally, macrophage polarisation showed the same results as in the small intestine, as a significant decrease was observed in the M1/M2 ratio of KI/w+M mice compared to both the KI/w and KI/KI+M groups, with no difference between KI/KI and KI/KI+M (Figure 5I).

## 4. Discussion

The present study sought to clarify the role of ILC3s in immunoregulation and tissue homeostasis of the small intestine, in particular in a state of intestinal inflammation caused by a Western-type diet. Miso was employed in order to induce the proliferation of ILC3s in the small intestine, specifically of their IL-22-producing anti-inflammatory phenotype. Miso is a popular traditional Japanese fermented food that has been reported to have beneficial effects on health, and it represents a source of SCFAs due to its fermentation of fibre mediated by the microorganism *Aspergillus oryzae* [35]. In order to observe the influence of miso specifically on ILC3s, a mouse model lacking the expression of RORγt (KI/KI) was employed and compared to heterozygous mice of the same mouse strain (KI/w).

First, it was necessary to confirm that miso supplementation is capable of increasing the concentration of SCFAs in the intestine. For this study, miso was administered ad libitum in addition to mouse feed (HFHSD) and water, rather than in specific amounts. For this reason, the difference in miso consumption levels was not investigated in this research. Nevertheless, the miso supplementation was successful in increasing the levels of the SCFAs acetate, butyrate, and propionate in all of the mice that received it as part of their diet.

Previous studies performed by this laboratory have shown that the consumption of miso has beneficial effects on glycaemic control in patients with T2D. In particular, daily consumption of miso soup was correlated with lower haemoglobin A1c (HbA1c), although this was only the case in women and not in men. Additionally, the study could not determine if this was caused by miso consumption alone or by a combination of different components of the patients’ diet [36]. In the current study, glucose tolerance and insulin tolerance were tested in order to assess whether this effect could be reproduced in mice. Indeed, glucose tolerance was improved by miso supplementation in the presence of functioning ILC3s, as blood glucose was lowered despite the HFHSD. However, this was not the case for insulin resistance, which showed no difference regardless of genotype and diet. Moreover, HbA1c levels were not analysed in the mice.

In addition to glucose levels, visceral fat accumulation has been reported to be influenced by miso consumption in both humans and mice, especially when combined with other traditional Japanese dietary habits [37]. In this study, mice that received miso supplementation showed a lower weight gain, although the difference was not considerable. However, the choice of the specific mouse strain used for this study (RORγt knockout) was an additional factor that influenced the body weight throughout the treatment. Due to the impaired immune regulation of double knockout mice, these animals are reported to be more susceptible to weight loss, infection, and inflammation, and have a higher mortality rate compared to wild-type mice [38]. This was also the case for the current study, as the mortality rate of the KI/KI mice was much higher compared to the KI/w ones from the breeding phase and until the end of the study, regardless of their diet. The body weight measurements throughout the eight weeks of treatments showed a higher increase in the KI/w mice compared to KI/KI, although the miso supplementation caused a minor decrease in both cases.

Mainly, miso caused significant changes in the intestinal barrier integrity in the presence of functioning ILC3s. Factors associated with better digestive and absorption capability, efficient tissue turnover, and sufficient mucus production benefitted from the miso supplementation, but not in the double knockout mice [32]. Of course, ILC3s are only one of the factors involved in these processes. As fermented foods are known to be beneficial for the gut microbiota, the addition of miso to the HFHSD likely affected the commensal bacteria and played a beneficial role in its richness and diversity [39]. Additionally, the increased expression of *Muc2* was positively influenced by the presence of butyrate in the intestine, in addition to the regulatory effects of ILC3s [40]. Nevertheless, ILC3-mediated immune regulation proved to be a determining component in the maintenance of the intestinal barrier.

Indeed, significant changes were observed in the inflammatory state of the small intestine between the different groups. On one hand, ILC1s were decreased by miso supplementation in the presence of functioning ILC3s, as well as the pro-inflammatory cytokines TNF-α and IL-1β. On the other hand, macrophage polarisation presented the M2 anti-inflammatory phenotype. Most importantly, the levels in IL-22 expression were significantly increased by miso supplementation, in spite of the fact that the number of ILC3s did not differ between groups. The combined results of ILC distribution analysis and cytokine expression quantification showed that SCFAs did not induce an increase in the abundance of ILC3s, but rather stimulated their function and led to a higher production of IL-22. For this reason, RORγt double knockout mice possessed the same number of ILC3s as heterozygous mice but were unable to increase the production of IL-22 in response to miso supplementation.

Finally, the status of the liver was also assessed in this study, as HFHSD can cause accumulation of fat in the liver and can lead to NAFLD. However, this study did not observe marked differences in the liver condition between the four groups. Liver weight was not affected by the different treatments, and fat deposition in the liver was similar in all groups. Additionally, none of the mice developed liver dysfunction, as both the NAFLD score and fibrosis stage were low in all subjects. As for the inflammatory state of the liver, macrophage polarisation displayed similar results to those of the small intestine, as there was a prevalence of M2 macrophages following miso supplementation in the presence of functioning ILC3s. However, this was the only similarity with the small intestine. In the liver, ILC1 distribution was affected by the genotype rather than the miso supplementation, as RORγt double knockout mice showed an increase regardless of the diet compared to heterozygous mice which displayed lower levels. Interestingly, ILC3 distribution was different between groups, in contrast with the small intestine. In particular, double knockout mice presented an increase compared to heterozygous mice. The quantification of pro- and anti-inflammatory cytokines may give more insight into how the different ILC distribution affected the inflammatory state in the liver. However, due to the time limit this was not possible to investigate in the current study.

In order to further investigate the hypothesis of the current study, additional testing can be performed with miso supplementation. Firstly, this study did not investigate the impact of different amounts of miso consumed by the mice, nor of different variations of miso paste. Secondly, the SCFA concentration was only quantified in the intestine but not in the serum or in the liver, nor in the miso water that was administered. Moreover, the main focus of this research was on the small intestine, and the role of ILC3s in the liver was not extensively investigated.

Furthermore, due to the difficulty in employing RORγt double knockout mice, a different model may be utilised. A possible option is that of inhibiting ILC3s in wild-type mice, which could decrease the interference created by the susceptibility of the mutated mice. Additionally, other fermented foods and probiotics may be employed in order to induce the increase in SCFAs in the small intestine. Furthermore, a treatment longer than eight weeks may be able to produce more significant differences, as the development of metabolic dysregulation is a slow process that involves numerous factors.

## 5. Conclusions

In conclusion, this study demonstrated that miso supplementation influenced several factors involved in inflammation, such as macrophage polarisation and cytokine expression, and in intestinal barrier integrity, such as epithelial and mucous layer maintenance. Additionally, the study showed that the number of ILC3s is not the key factor in immune regulation, but rather the capacity of ILC3s to produce IL-22 and employ it to control the immune response in the small intestine.

## Data Availability

All data are available upon reasonable request to the corresponding author.

## Supplementary Materials

The following supporting information can be downloaded at: https://www.mdpi.com/article/10.3390/nu16213743/s1, Figure S1: Strategy for innate lymphoid cells (ILCs). Figure S2: Strategy for macrophages.
