# Supplementary figures and images for: Supplementation of Miso to a Western-Type Diet Stimulates ILC3s and Decreases Inflammation in the Small Intestine
Source: Nutrients. 2024 Oct 31;16(21):3743. doi: 10.3390/nu16213743 (PMC11547460; doi:10.3390/nu16213743)

Supplementary Figure 1

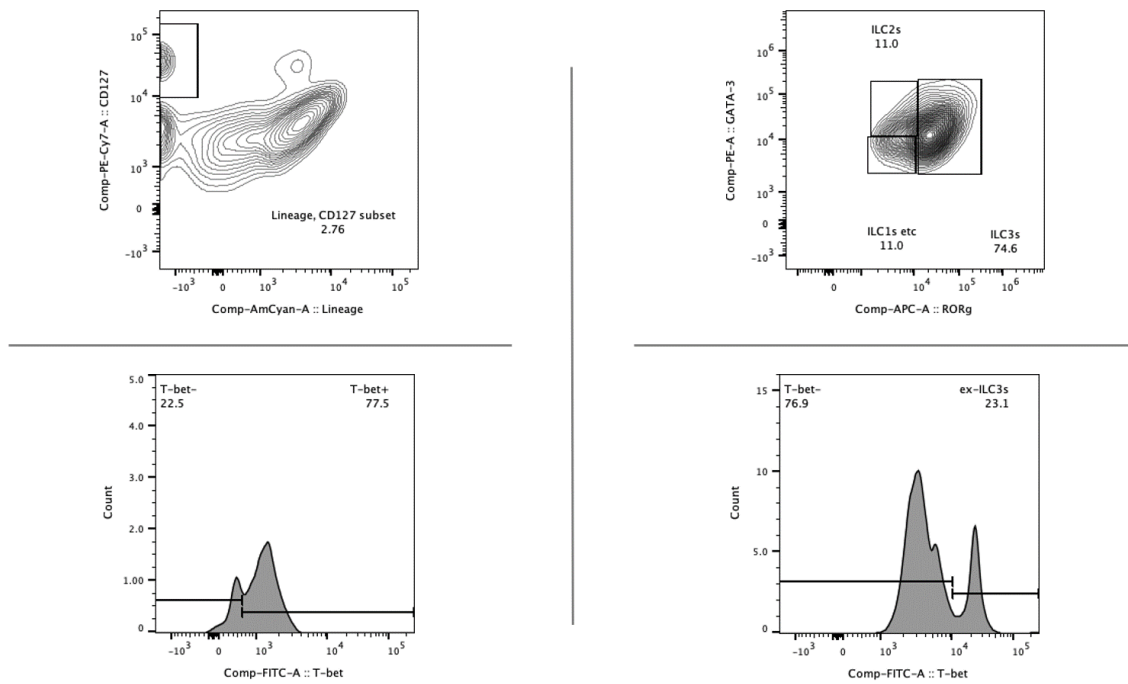

Supplementary Figure 2

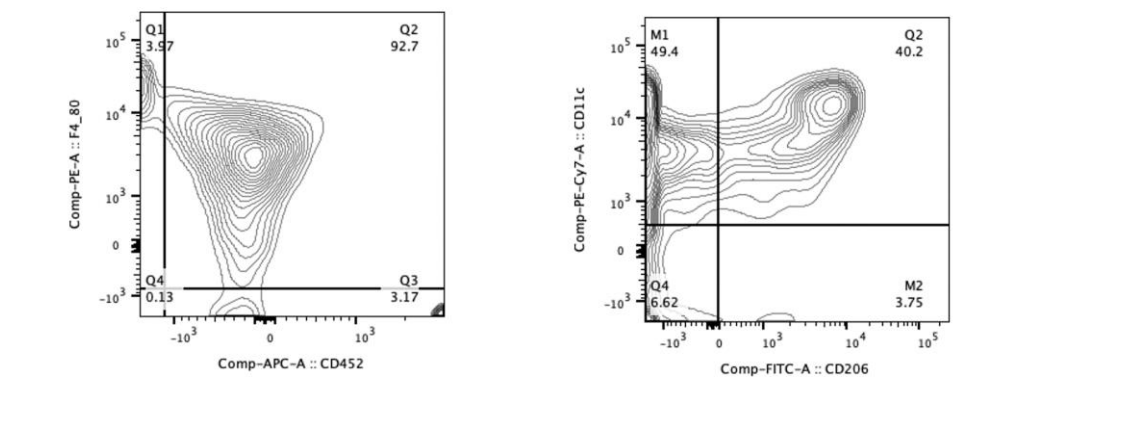

Supplement: Supplementary file 1 [file nutrients-16-03743-s001.zip › nutrients-3261038-supplementary.pdf]
